# Detection of probable dementia cases in undiagnosed patients using structured and unstructured electronic health records

**DOI:** 10.1186/s12911-019-0846-4

**Published:** 2019-07-09

**Authors:** Yijun Shao, Qing T. Zeng, Kathryn K. Chen, Andrew Shutes-David, Stephen M. Thielke, Debby W. Tsuang

**Affiliations:** 1 George Washington University, 800 22nd St. NW, Science and Engineering Hall, Ste. #8390, Washington, DC 20052 USA; 2 Washington DC VA Medical Center, 50 Irving St. NW, Washington, 20422 DC USA; 3 Geriatric Research, Education, and Clinical Center, S182 GRECC, VA Puget Sound Health Care System, 1660 S. Columbian Way, Seattle, WA 98108 USA; 4 Mental Illness Research, Education, and Clinical Center, S116 MIRECC, VA Puget Sound Health Care System, 1660 S. Columbian Way, Seattle, WA 98108 USA; 5 Department of Psychiatry and Behavioral Sciences, University of Washington, 1959 NE Pacific St., Box 356560, Seattle, WA 98195 USA

**Keywords:** Dementia, Diagnosis, Machine learning, Medical records, Veterans

## Abstract

**Background:**

Dementia is underdiagnosed in both the general population and among Veterans. This underdiagnosis decreases quality of life, reduces opportunities for interventions, and increases health-care costs. New approaches are therefore necessary to facilitate the timely detection of dementia. This study seeks to identify cases of undiagnosed dementia by developing and validating a weakly supervised machine-learning approach that incorporates the analysis of both structured and unstructured electronic health record (EHR) data.

**Methods:**

A topic modeling approach that included latent Dirichlet allocation, stable topic extraction, and random sampling was applied to VHA EHRs. Topic features from unstructured data and features from structured data were compared between Veterans with (*n* = 1861) and without (*n* = 9305) ICD-9 dementia codes. A logistic regression model was used to develop dementia prediction scores, and manual reviews were conducted to validate the machine-learning results.

**Results:**

A total of 853 features were identified (290 topics, 174 non-dementia ICD codes, 159 CPT codes, 59 medications, and 171 note types) for the development of logistic regression prediction scores. These scores were validated in a subset of Veterans without ICD-9 dementia codes (*n* = 120) by experts in dementia who performed manual record reviews and achieved a high level of inter-rater agreement. The manual reviews were used to develop a receiver of characteristic (ROC) curve with different thresholds for case detection, including a threshold of 0.061, which produced an optimal sensitivity (0.825) and specificity (0.832).

**Conclusions:**

Dementia is underdiagnosed, and thus, ICD codes alone cannot serve as a gold standard for diagnosis. However, this study suggests that imperfect data (e.g., ICD codes in combination with other EHR features) can serve as a silver standard to develop a risk model, apply that model to patients without dementia codes, and then select a case-detection threshold. The study is one of the first to utilize *both* structured and unstructured EHRs to develop risk scores for the diagnosis of dementia.

## Background

Dementia significantly decreases quality of life and increases inpatient service utilization [1, 2], outpatient mental health visits, and health-care costs, both in civilian contexts [3, 4] and within VHA [1]. Many of these consequences can be at least moderately reduced when dementia is identified earlier in the course of illness. For instance, patients with dementia can benefit from liaisons to mental-health care providers, as well as social and/or legal services related to elder and dementia care. Providing access to these resources can delay nursing home placement and thereby increase the ability for patients to age in place, which can improve quality of life and generate substantial health-care savings. Although the US Preventive Services Task Force does not recommend routine screening for dementia because of the high rate of false positives in such screenings [1, 5], the identification of and appropriate workup for dementia is critical for chronic disease management and health-system capacity planning.

The estimated prevalence of dementia in Veterans over the age of 65 is 7% (ranging from 5.8 to 9.4%) [1], rising to 24% in Veterans over the age of 85 and then to 30% in Veterans over the age of 95 [6]. The underdiagnosis of dementia, however, is common [7]. A recent study by Amjad et al. [8], for instance, concluded that only about half of individuals who meet criteria for dementia actually receive such a diagnosis from a clinical provider, and Butler et al. [9] found that among Veterans who receive a diagnosis of dementia, at least one-third receive a nonspecific “dementia, not otherwise specified” diagnosis when a more specific diagnosis would be more appropriate. These trends are not surprising when we consider the complexity of making a dementia diagnosis and the fact that modern medicine relies on self-management and self-reliance, traits that are severely diminished by cognitive impairment or dementia. The lack of consistency in identifying, working up, and diagnosing dementia reduces the utility of using diagnostic codes and other administrative data, increases the health-care costs described above, and points to a missed opportunity to improve quality of life and delay institutionalization by providing potentially beneficial dementia-related services, outpatient medical care, and medications [10, 11]. New approaches to facilitate the timely detection of dementia in primary-care settings are thus needed to address the quality of care and to provide adequate health-care resources to individuals with dementia.

Electronic health records (EHRs) offer a rich resource and new opportunity to improve research and clinical care, particularly in the context of undiagnosed dementia. Structured EHR data, such as diagnosis and procedural codes, have been used extensively in population-based research [12], the identification of specific patient characteristics, the performance of surveillance, and the establishment of risk scores [13]. Multiple research consortia, including the Electronic Medical Records and Genomics Network (eMERGE) [14], have used EHRs to construct and identify phenotypes. One of the eMERGE studies created a model that used ICD-9 diagnostic codes and the occurrence of “events” (i.e., neuroimaging, orders for B12 or TSH) and pharmacy fills for dementia medications to better identify the dementia phenotype in EHRs [15]. The eMERGE approach, which identified the best criterion for identifying dementia as “all cause dementia” (i.e., > 5 ICD-9 codes for dementia) and/or > 1 pharmacy fill for a dementia medication, had a sensitivity of 55% and a positive predictive value of 73%. A subsequent family practice–based health study of administrative data [16] identified the highest performing algorithm as one that included a hospitalization code, three physician claim codes that occurred at least 30 days apart, or the prescription of an Alzheimer’s disease (AD)–related medication.

However, algorithms that focus on structured EHR data and rely on diagnostic codes are unable to reliably identify conditions that are not diagnosed formally. Indeed, the fact that many cases of dementia and other cognitive impairment are not documented in administrative or diagnostic codes indicates that such codes alone are insufficient in identifying cases. To validate diagnosis would at a minimum require the manual review of potential cases for signs of cognitive impairment by a dementia specialist, but reading every patient note in search of memory or daily functioning difficulties is clearly not practical.

To address these obstacles, we sought to develop an efficient phenotyping method that would incorporate the analysis of both structured EHR data (e.g., diagnosis and procedural notes) and unstructured EHR data (e.g., words in clinical notes). A key challenge to this strategy, especially for undiagnosed dementia, is the highly varied documentation of the clinical presentation of dementia in clinical notes and the lack of expert annotated data. Indeed, other investigators have had promising results when attempting to detect dementia using both structured and unstructured data [17, 18], but these past efforts, while sophisticated and informative, analyzed subjects who received specific cognitive disorder diagnoses (and excluded undiagnosed subjects) per Alzheimer’s Disease Neuroimaging Initiative (ADNI) criteria. Furthermore, these analyses of unstructured data were only conducted in clinical research records—not in EHRs. In contrast, EHR-based detection, which is more translatable to clinical practice at VHA, must focus on learning to detect undiagnosed dementia from a large amount of imperfect data and such detection is potentially more challenging.

Thus, the creation of a reliable dementia phenotype classifier through machine learning requires a large sample with annotation. In VHA, we have access to a tremendous amount of data that we believe can allow us to develop a silver standard for the diagnosis of dementia. This data contains a large number of Veterans who received dementia diagnoses within VHA and who mostly likely *do* have dementia (i.e., they are true positives); a large number of Veterans who have not received dementia diagnoses and who most likely do *not* have dementia (i.e., they are true negatives); and a large number of Veterans who have not received dementia diagnoses but do have dementia (i.e., they are false negatives). In fact, we suspect that among Veterans with dementia about 30 to 50% are in the latter category. Leveraging this large body of data, we sought to design a weakly supervised learning approach and to validate the machine-learning results through manual chart reviews of a subset of subjects by dementia specialists. We hypothesized that documented signs of dementia would be found in the imperfect EHR data of VHA patients who lacked a dementia diagnosis.

## Methods

### Study population

For this study, we created cohorts of Veterans with and without a diagnosis of dementia who were previously evaluated at VA Puget Sound. The dementia cases were Veterans who

1. received at least one diagnosis of dementia from a specialty clinic as defined by one of the following ICD-9 codes: 331.0, 290.0, 290.10, 290.11, 290.12, 290.13, 290.20, 290.21, 290.3, 290.40, 294.10, and 294.11;
2. received their first dementia diagnosis at an age of 65 or older and between FY2009 and FY2014; and
3. had at least 2 days per year with documented clinical visits and associated notes in Computerized Patient Record System (CPRS) for each of the 3 years preceding the first dementia diagnosis.

We intentionally focused on cases with ICD codes established in specialty clinics because past studies have shown that a dementia diagnosis made by specialty clinicians are highly specific [9]. The controls were Veterans who

1. had at least one outpatient visit or inpatient hospitalization at VA at an age of 65 or older;
2. received no dementia-related diagnoses (see the ICD-9 codes above) over a period of 3 years; and
3. were not prescribed anti-dementia medications (i.e., donepezil, galantamine, rivastigmine, or memantine) over the three-year analysis period.

The controls were matched to dementia cases (5:1) on gender, age (within 5 years), and Charlson comorbidity index (CCI) [19] as a way to reduce the contributions of these variables to the differences that might be observed in structured and unstructured data between cases and controls. We did not expect that the CCI would control for all possible confounding comorbidity-related variables; instead, we sought to reduce the contributions of *overall* medical comorbidity severity.

### Data source

For both cases and controls, we obtained structured data (i.e., diagnosis [ICD codes], procedures [CPT codes], medications, and clinical document types) and unstructured data (i.e., clinical document text) from the clinical data warehouse (CDW) within the Veterans Affairs Informatics and Computing Infrastructure (VINCI), which was established to improve researchers’ access to VHA data and to facilitate the analyses of these data while also ensuring Veterans’ privacy and data security. This resource comprises clinical and administrative domains, including notes, on 25 million patients.

All clinical data were collected for a 3-year period that either immediately preceded but did not include the first ICD-9 diagnosis of dementia (for cases) or a random visit date that was selected as an index date (for controls). This 3-year period was established to capture data from potentially symptomatic cases who were receiving medical care at VHA prior to their diagnosis.

Medical comorbidities were gathered from administrative data, particularly from ICD codes assigned at admission and outpatient visits. The comorbidities were then assessed using the CCI [19], and each subject was assigned a comorbidity category (i.e., 0, 1–2, 3–4, or > 4) based on the initial description of Deyo et al.; weights were applied as initially described by Deyo et al. [19]. Age was calculated at the first ICD-9 dementia diagnosis for cases or at the pre-selected index date (i.e., the latest visit) for controls.

### Structured data aggregation

We aggregated the structured data over the 3-year analysis period, treating each type of structured data (i.e., an ICD or CPT code, medication, or note type) as a candidate feature. The prevalence of the candidate features in the case and control samples was then calculated and used for feature selection.

### Topic modeling and stable topic extraction

We used a topic modeling approach to identify findings related to dementia in the free-text clinical notes, as we previously described in Shao et al. [20]. Topic modeling is an unsupervised machine-learning method for automatically discovering common themes, called “topics,” that are shared by documents in a large text corpus. These topics are technically represented as a series of words that frequently co-occur in documents. The number of topics is usually a few orders less in magnitude than the number of documents; this makes understanding the content of a large text corpus easier because one can analyze the smaller number of topics rather than reading the larger number of documents. Our topic modeling method also calculates the proportion of topics in each document, and these calculations make it feasible to automatically retrieve documents that are relevant to certain themes. In this study, for example, we use the proportion of dementia-related topics observed in excess in cases versus controls to identify dementia-related signs or symptoms.

To ensure that the topics identified by our model were stable, we used a two-step topic modeling approach [20]. First, to discover raw topics, we ran a latent Dirichlet allocation (LDA) algorithm on the clinical notes. This algorithm was implemented in the Machine Learning for Language Toolkit (MALLET) Java package. Because LDA uses a randomize seed, the resulting topics differ slightly in each run. Thus, we ran LDA three times to obtain three sets of raw topics. In preparation for the next step, we then applied a stable topic extraction method [20] to the 3 sets of raw topics to extract the topics that are stable. Given that we collected about 2.5 million notes from cases and controls during the 3-year study period and that LDA is a time-consuming algorithm, we randomly sampled 1 note per day for each subject; this sampling strategy reduced the runtime of the LDA algorithm while preserving the main topics. This yielded a sufficiently large and representative sample corpus of 871,000 notes. We then ran LDA on the sampled notes and set 1000 as the total number of topics. We ran LDA three times to obtain 3 topic models, and then we applied the 3 models to all of the 2.5 million notes using the LDA inference algorithm to infer the topic proportions in each note.

Second, to extract stable topics from the set of raw topics, we applied a stable topic extraction method to the 3 sets of raw topics [20]. The application of this step yielded 877 stable topics. We then determined whether these stable topics were present in each of the notes by calculating whether at least 2 of the 3 topic proportions (i.e., one proportion value from each run) were ≥ 2%; the value 2% was an empirical choice. We considered that a topic was present in a subject if it was present in at least one of the notes of that subject. These topics were directly used to form features from unstructured data.

### Feature selection

We extracted thousands of structured data features (i.e., ICD and CPT codes, medications, and document types) and hundreds of topic features. By comparing the features present in the cohort of Veterans with an ICD-9 diagnosis of dementia (*n* = 1861) to the features present in the cohort of Veterans without an ICD-9 diagnosis of dementia (*n* = 9305), we were able to select features that were highly associated with the diagnosis of dementia. Specifically, we calculated the correlation and odds ratio (OR) of each feature associated with case-control status, and we selected topics that were present in > 1% of the case or control records that were either correlated to case-control status (i.e., a correlation > 0.05 or < − 0.05) or to an OR of > 2.0 or < 0.5 (before adjustment).

### Dementia risk score and the identification of undiagnosed dementia

We created a logistic regression model using the selected features as predictors. We categorized all cases and controls by their corresponding logistic regression prediction scores, which we considered their “risk” scores. Given the high rate of undiagnosed dementia in VHA, risk scores for controls (i.e., subjects without a dementia diagnosis) were especially relevant. For example, controls with regression prediction scores that are highly associated with dementia may benefit from additional workup.

When we fit the logistic regression model, we used a value of 1 for the outcome of the cases and 0 for the outcome of the controls such that higher prediction scores indicate a higher likelihood of having dementia. The controls with high risk scores were thus identified as undiagnosed dementia by the model. To define “high” risk scores, we introduced a threshold *θ* and defined high scores to be those > *θ*. In other words, controls with scores > *θ* were identified as having undiagnosed dementia by the model. Using a variable threshold *θ* provided us with the flexibility to choose identifications with different performance characteristics (i.e., some for higher sensitivity and some for higher specificity).

### Validation

To validate the risk scores and choose a threshold *θ*, a reference standard is required. We first confirmed that there were sufficient data within the free-text notes for trained clinicians to independently assign dementia diagnoses and to achieve an adequate level of inter-rater agreement between each other. To that end,10 cases and 10 controls were randomly selected for manual record review by two dementia specialists (DWT and KKC) who were blinded to subjects’ case-control status. For these 20 subjects, the specialists reviewed a total of 2092 free-text clinical notes that were dated within the 3-year window prior to either the first dementia diagnosis date (of the cases) or the index date (of the controls); the specialists determined the presence of dementia using DSM-V guidelines [21]. The clinicians demonstrated high inter-rater agreement, and the agreement between the ICD-9 dementia diagnoses and the clinician-assigned diagnoses was also high (Kappa = 0.810, 95% confidence interval 0.571, 1.0), which is consistent with a previous study [9]. The clinicians also found that *all* 10 of the cases had early signs of dementia and that a few of the controls actually had undiagnosed dementia.

To evaluate the predicted risk scores for controls (i.e., the subjects without ICD codes of dementia), we established 10 risk bins such that bin 1 was designated for risk scores between 0 and 0.1, bin 2 was designated for risk scores between 0.1 and 0.2, and so on until bin 10, which was designated for scores between 0.9 and 1. We then randomly selected 10 controls from each of these 10 risk bins, as well as an additional 20 subjects from the low risk-score bin (i.e., 30 controls were selected from bin 1), as this bin had several times more subjects without a dementia diagnosis than the other bins. This stratified sampling was used so that controls with a full range of risk scores could be reviewed by our dementia specialists in a small validation sample. However, because the subjects for the validation sample were selected from bins that varied in size, the calculation of sensitivity (SEN) and specificity (SPE) is less straightforward than if each of the risk bins contained an equal number of subjects.

Following the diagnostic guidelines described above, the two dementia specialists (DWT and KKC) reviewed a total of 22,980 clinic notes from these 120 controls to determine whether each control demonstrated signs and symptoms in the three-year analysis period that were consistent with “Dementia” or “Non-Dementia.” To speed up this manual review process, the specialists generated a list of dementia keywords (e.g., *memory* and *cognitive*) that were then highlighted in the notes. Some of the subjects lacked sufficient information for the specialists to determine their dementia status; these subjects were categorized as “Unclear” and were subsequently treated as “Unclear = Dementia” or “Unclear = Non-Dementia” in different analyses (e.g., see Table 3).

A linear model was fit to the rates of undiagnosed dementia as determined by manual chart review for each risk bin. This approach was used because (1) the selection of thresholds requires more granular estimates of undiagnosed dementia rates while the cutoffs used for the 10 preset bins are coarse, and (2) the variance of estimation with a fitted line is greatly reduced compared to using individual bins with very small sample sizes (i.e., *n* = 10 or *n* = 30 per bin). Given that linear regression models require each rate to correspond to a single x-value rather than to an interval, we chose the midpoints of each bin interval as the x-values. For example, for the 0-to-0.1 bin, we set the x-value at 0.05.

Line fitting was performed under the hypothesis that the intercept was zero. That is, we hypothesized that when the prediction score decreased to zero, the incidence rate of dementia would decrease to zero as well. To test the hypothesis that the intercept was zero, we first fit a line with a non-zero intercept: *y* = *b*_0_ + *b*_1_*x*. The regression results showed that the *p*-value for *b*_0_ was > 0.05, which meant that we could not reject the hypothesis that *b*_0_ = 0. Therefore, we fit a second line with a zero intercept: *y* = *bx*. This fitted line was used to estimate the rates of undiagnosed dementia in arbitrary bins.

To create finer-grained bins with even sizes, we sorted all of the controls by their risk scores and divided them into many bins such that each bin contained ~ 100 controls; a total of 9305 controls were divided into 93 bins. Assuming that 0 = *x*_0_ < *x*_1_ < *x*_2_ < ⋯ < *x*_92_ < *x*_93_ = 1 were the risk score values that divided the controls into the 93 bins, the number of undiagnosed dementias in the *i* th bin *x*_*i* − 1_~*x*_*i*_ was estimated to be

$$ {u}_i=\mathrm{round}\left(b\bullet {\overline{x}}_i\bullet {N}_i\right) $$

where *b* is the slope from the fitted line, *y* = *bx*, ${\overline{x}}_i=\left({x}_{i-1}+{x}_i\right)/2$ is the midpoint of the bin, *x*_*i* − 1_~*x*_*i*_, *N*_*i*_ is the actual number of controls in the bin (*N*_*i*_ ≈ 100), and the function round(∙) rounds any value to the nearest integer.

The SEN and SPE could only be estimated if the threshold *θ* was set to be one of the dividing values *x*_*i*_. Thus, for threshold *θ* = *x*_*i*_,

$$ \mathrm{SEN}=\frac{\sum_{k=i+1}^{93}{u}_k}{\sum_{k=1}^{93}{u}_k},\kern2.75em \mathrm{SPE}=\frac{\sum_{k=1}^i\left({N}_k-{u}_k\right)}{\sum_{k=1}^{93}\left({N}_k-{u}_k\right)}=\frac{\sum_{k=1}^i{N}_k-{\sum}_{k=1}^i{u}_k}{9305-{\sum}_{k=1}^{93}{u}_k} $$

By varying the threshold *θ* from *x*_0_ to *x*_93_, we were able to plot the receiver of characteristic (ROC) curve and calculate the area under the ROC curve (AUC).

## Results

Table 1 describes the demographic characteristics of the cases and controls that were utilized for the analyses in this study. Given that we matched the cases and controls on age and gender, there should be no differences between the groups on these characteristics.

**Table 1:** Demographics of the cases and controls

	Cases (*n* = 1861)	Controls (*n* = 9305)
Mean age	79.8	79.5
Gender		
Female	62 (3.3%)	310 (3.3%)
Male	1799 (96.7%)	8995 (96.7%)
Race		
Black	112 (6.0%)	428 (4.6%)
White	1434 (77.1%)	7099 (76.3%)
Other	64 (3.4%)	245 (2.6%)
Unknown	251 (13.5%)	1533 (16.5%)
Ethnicity		
Hispanic	28 (1.5%)	135 (1.5%)
Non-Hispanic	1679 (90.2%)	8170 (87.8%)
Unknown	154 (8.3%)	1000 (10.7%)

A total of 853 features were selected, including 290 topics, 174 non-dementia ICD codes, 159 CPT codes, 59 medications, and 171 note types. For example, a topic containing the terms *dementia*, *memory*, *cognitive*, *Alzheimer*, *MMSE*, and *recall* was selected, and that topic occurred in the notes of 74.94% of cases prior to ICD-9 dementia diagnosis but only in the notes of 13.51% of controls prior to the index time point (OR = 19.15).

The most significant topic features are shown in Table 2. In considering these results, note that (a) the terms in a topic could occur in any order or combination and (b) the presence of a topic in a document does not require the presence of all the terms in a topic to be present. Topics that were observed more frequently in cases than in controls were considered dementia related.

**Table 2:** The most significant topic features (*p* < 0.01) between cases and controls

#	Topic (showing 10 of the most *common* words in a topic)
1	dementia, memory, cognitive, wife, problems, loss, impairment, galantamine, mmse, Alzheimer, …
2	angry, asked, behavior, police, upset, told, staff, agitated, made, leave, ...
3	family, home, daughter, care, member, members, sister, granddaughter, grandson, brother, ...
4	qd, bid, prn, mg, qhs, lisinopril, tid, asa, metoprolol, meds, ...
5	plan, agree, reviewed, note, examined, discussed, findings, assessment, resident, concur, ...
6	ct, scan, contrast, chest, radiology, abdomen, pelvis, ordered, cat, pet, ...
7	taking, meds, pills, medication, takes, stopped, states, prescribed, pill, tabs, ...
8	resident, care, visit, nursing, home, staff, contract, daily, offered, date, ...
9	issues, related, health, problems, medical, issue, discussed, time, plan, treatment, ...
10	transfer, patient, report, transferred, ward, care, receiving, rn, condition, unit, ...
11	continues, continue, reports, remains, continued, time, encouraged, work, plan, improved, ...
12	housing, stable, months, part, stay, living, worried, household, rent, past, ...

**Fig. 1:**
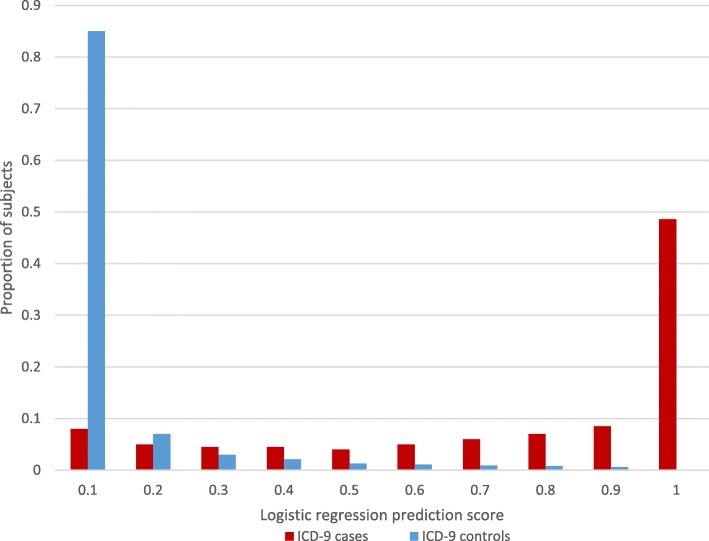
Prediction scores and originally assigned case/control status. The distribution of cases (red bars) and controls (blue bars) established by our original inclusion and exclusion criteria compared to the results of our logistic regression model (prediction score indicates the likelihood of having dementia)

Comparing the distribution of cases and controls in our logistic regression model to the distribution of subjects as established by our original inclusion and exclusion criteria (i.e., ICD-9 diagnosis of dementia) shows that the majority of controls had low risk scores, and the majority of cases had high risk scores (see Fig. 1).

In Table 3, we list the results of our chart review on 120 subjects from the control group (as described in the Validation subsection above). We also depict our calculation of the rate of undiagnosed dementia for each risk-score bin.

**Table 3:** Results of the manual chart review

Risk score bin	# of dementias	# of unclears	# of non-dementias	Rate of undiagnosed dementia^a^	
				Unclear = dementia	Unclear = non-dementia
0.0 ~ 0.1	3	1	26	0.133	0.1
0.1 ~ 0.2	2	1	7	0.3	0.2
0.2 ~ 0.3	2	0	8	0.2	0.2
0.3 ~ 0.4	6	1	3	0.7	0.6
0.4 ~ 0.5	3	2	5	0.5	0.3
0.5 ~ 0.6	5	0	5	0.5	0.5
0.6 ~ 0.7	5	1	4	0.6	0.5
0.7 ~ 0.8	6	0	4	0.6	0.6
0.8 ~ 0.9	8	0	2	0.8	0.8
0.9 ~ 1.0	7	2	1	0.9	0.7

^a^“Unclear = dementia” indicates that subjects who were classified as “unclear” during the manual chart review are classified in the “dementia” group and the rate of undiagnosed dementia is calculated using this formula: (# of dementias + # of unclears) / (# of dementias + # of unclears + # of non-dementias). Conversely, “Unclear = non-dementia” indicates that subjects who were classified as “unclear” during the manual chart review are classified in the “non-dementia” group and the rate of undiagnosed dementia is calculated using this formula: (# of non-dementias + # of unclears) / (# of dementias + # of unclears + # of non-dementias)

**Fig. 2:**
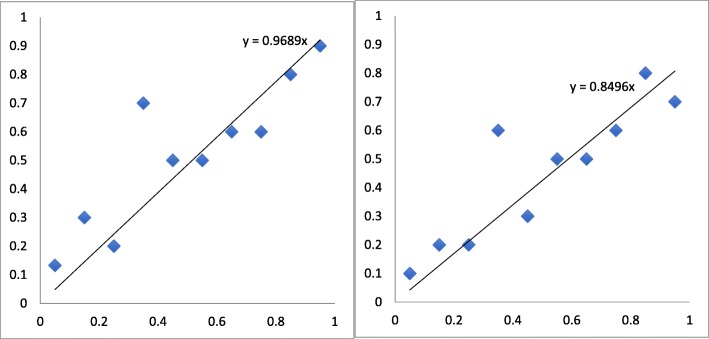
Lines fit to the rates of undiagnosed dementias estimated in the 10 risk bins. The x values for the bins were taken as the midpoints of the bin intervals. The left figure illustrates the results when the “Unclear” diagnoses were treated as dementia (i.e., “Unclear = Dementia”), whereas the right figure illustrates the results when the “Unclear” diagnoses were treated as non-dementia (i.e., “Unclear = Non-Dementia”)

We fit a linear regression model on the rate of undiagnosed dementia vs. risk score based on the calculations in Table 3, as depicted in the two rightmost columns. The risk score for each bin was taken as the midpoint. The regression results are shown in Table 4. The *p*-values there indicated that the intercept values were not significantly different from zero (at the 0.05 level), and we therefore fit a second linear regression model with intercept = 0. The data points and the fitted lines from this second regression model are shown in Fig. 2.

**Table 4:** Linear regression results

	Unclear = Dementia^a^		Unclear = Non-Dementia^a^	
	Intercept	Slope	Intercept	Slope
Value	0.1565	0.7335	0.1015	0.6970
*p*-value	0.084	0.001	0.199	0.001

^a^“Unclear = dementia” indicates that subjects who were classified as “unclear” during the manual chart review are classified in the “dementia” group, whereas “Unclear = non-dementia” indicates that subjects who were classified as “unclear” during the manual chart review are classified in the “non-dementia” group

**Fig. 3:**
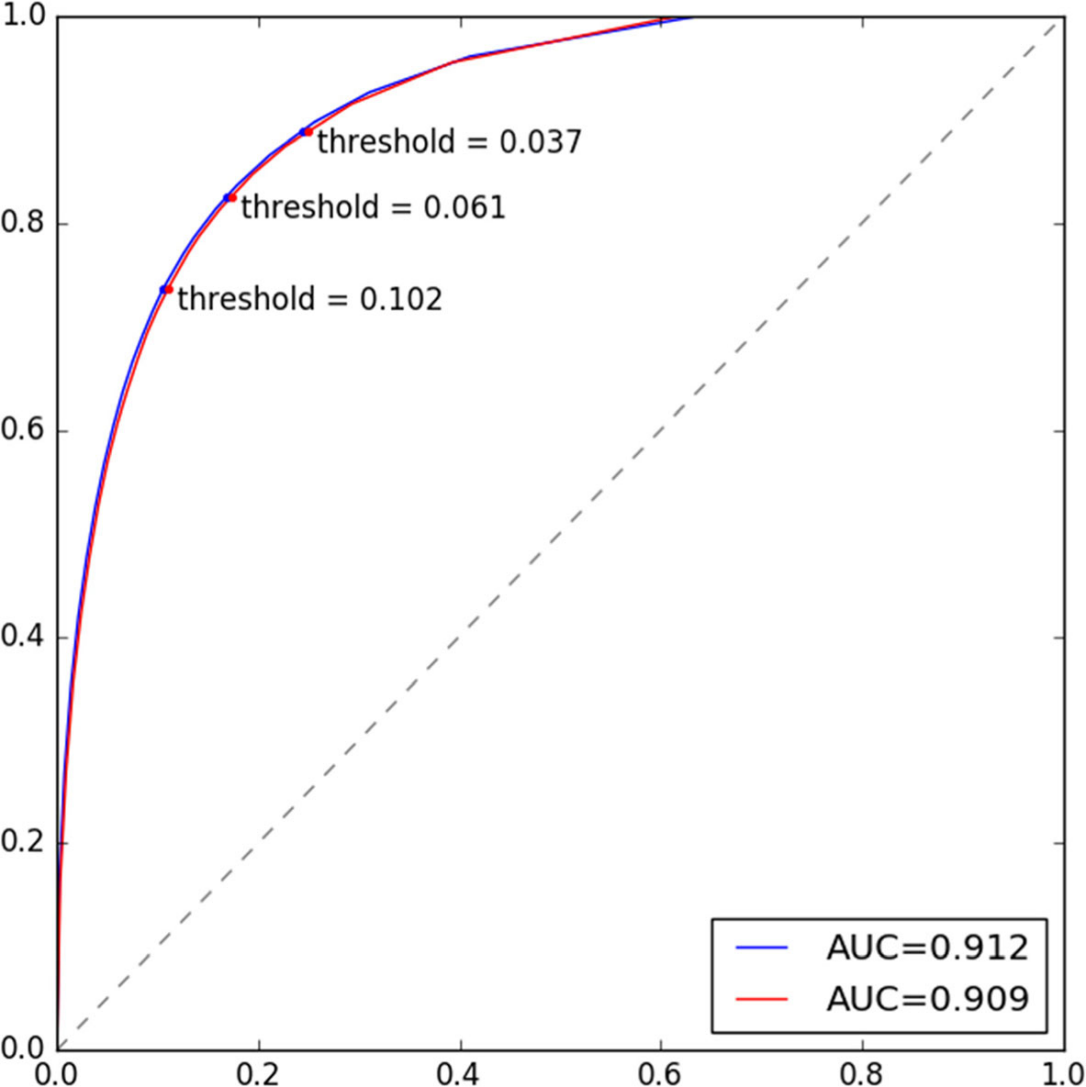
ROC curves of the prediction with AUC values. The blue and red colors correspond to “Unclear = Dementia” and “Unclear = Non-Dementia,” respectively. AUC: area under ROC; ROC: receiver of characteristic curve

Figure 3 shows the ROC curves of the model, which draws from 93 bins and uses the fitted linear regression model with zero intercept for estimating the number of dementias within each bin, in identifying undiagnosed dementia when the “Unclear” diagnoses were treated as dementia (in blue) and non-dementia (in red). The AUCs were 0.912 and 0.908, respectively. In the figure we have highlighted six dots (i.e., 3 blues and 3 reds) that correspond to 3 thresholds; these dots represent 3 typical situations of the performance: threshold = 0.037 had higher sensitivity, threshold = 0.102 had higher specificity, and threshold = 0.061 had balanced sensitivity and specificity. Table 5 lists the specific sensitivity and specificity values for the 3 thresholds.

**Table 5:** Performance for the identification of undiagnosed dementias

Threshold	Unclear = Dementia^a^		Unclear = Non-Dementia^a^	
	SEN	SPE	SEN	SPE
0.037	0.889	0.756	0.888	0.751
0.061	0.825	0.832	0.826	0.827
0.102	0.736	0.895	0.736	0.890

SEN: sensitivity; SPE: specificity. ^a^ “Unclear = dementia” indicates that subjects who were classified as “unclear” during the manual chart review are classified in the “dementia” group, whereas “Unclear = non-dementia” indicates that subjects who were classified as “unclear” during the manual chart review are classified in the “non-dementia” group

## Discussion

Our study is the first step toward establishing a model to detect probable dementia cases among patients who have not received a dementia-related diagnosis or ICD code. Because of the high underdiagnosis rate of dementia, we cannot rely on ICD codes as a gold standard. However, our findings suggest that we can use imperfect data (e.g., the ICD codes in combination with other EHR features) as a silver standard to develop a risk model, apply that model to patients without a dementia diagnostic code, and then select a threshold for case detection. This finding is particularly useful given the flaws inherent to other methods of identifying undiagnosed dementia. Broad-based dementia screening programs, for example, have not been widely adopted in clinical settings, in part because when implemented, such screening programs have been associated with high false-positive rates, patient hesitation to undergo diagnostic confirmation, and a high monetary cost per identified case [22, 23]. Older adults are particularly wary of the implications and potential psychological harms of dementia screening, such as the placement of more restrictions on their daily lives if dementia is diagnosed [24–26]. Given these factors, as well as the lack of an objective diagnostic test for dementia or the existence of specific medications to cure dementia, cognitive screening programs have been a low priority for both researchers and clinicians.

An alternative to systematic screening is a case-finding approach in which clinicians initiate a diagnostic assessment of dementia when patients (and/or their caregivers) describe or present with symptoms that are suggestive of dementia. However, these kinds of case-finding approaches are also flawed, as individuals with signs of dementia are often missed in primary care practice, both in civilian contexts [27–29] and within VHA [30]. In fact, some studies indicate that only ~ 25–40% of patients with dementia are recognized in primary care settings, and most surprising of all, this trend can sometimes include cases who are late in the disease course [29, 31]. Multiple factors contribute to the tendency of providers to overlook dementia cases, including time and resource constraints, a lack of objective measurements, the insidious onset of dementia symptoms, and the erroneous belief that there are no approved treatments [32–34].

A recent study found that using EHRs in combination with brief telephone-based cognitive screening assessments and follow-up calls resulted in up to seven times more diagnoses of dementia than in age-matched comparison Veterans [35]. Here, we follow the trajectory of those findings by proposing an automated EHR approach to improve case-finding in primary care. Our findings show that there are terms in notes and coded EHR data that are more likely to be associated with dementia cases than controls, and our examination of these terms suggests a high rate of undiagnosed dementia in VHA. We also found that these dementia-related word topics, non-dementia ICD-9 codes, procedure codes, specific medications, and visit types were documented in EHRs many months—and, in some instances, years—prior to subjects’ initial ICD-9 dementia diagnoses. Although the ICD-9 diagnoses of dementia that were assigned in EHRs were generally accurate according to our limited clinical review, the undiagnosed cases present significant clinical implications for resource planning in large health-care organizations like VHA.

Therefore, rather than relying on primary care providers or caregivers to initiate diagnostic assessments, automated case-finding algorithms could be implemented in clinics with a high number of geriatric patients (i.e., patients who have a high risk of developing dementia). We do not propose these algorithms as a method of generating a clinical diagnosis of dementia or as a substitute for an expert clinical assessment but, rather, as a possible method to flag patients who may benefit from a targeted clinical assessment. This alternative approach could result in earlier identification of patients with dementia, leading to more timely interventions (e.g., the prescription of anti-dementia medications and/or the involvement of social services) to potentially decrease morbidity. Indeed, since dementia patients have higher levels of medical comorbidity, they will need additional supports and resources to improve their and their families’ ability to manage these complicated medical comorbidities. Appropriate interventions will also improve their quality of life.

Although Wray et al. [35], and others, have previously used structured EHR data to assist in the diagnosis of dementia, and Bullard et al. [17, 18] have used structured and unstructured data from research records in a smaller sample, ours is the first study of EHRs to explore the utility of weakly supervised learning and natural language processing (NLP) in patients without the diagnosis of dementia. Our study is also unique in that we focused on EHR data present up to 3 years prior to the first ICD-9 diagnosis of dementia. As we demonstrate here, the use of our model to detect probable dementia cases who did not receive ICD codes can result in important increases in the early detection of dementia. Because of the high rate that dementia goes undiagnosed by clinicians, we cannot use ICD codes as a *gold standard* for diagnosis and optimize a predictive model based on ICD codes. However, our findings show that we *can* use existing EHRs to develop a risk model that can then be applied to individuals without dementia diagnostic codes. By applying the method as we have outlined it in this manuscript, it may be possible to focus automated screening, for example, on 2% of older patients (i.e., patients who are age > 65) who do not have a dementia diagnosis and then to offer additional workup.

Future studies that investigate the use of automated methods to detect undiagnosed dementia should consider applying weakly supervised machine learning to broader populations, expanding the validation stage, and gathering caregivers and primary-care providers’ insight on how to handle the risks of dementia that are suggested by algorithms. There are several limitations to this study in that we applied our algorithm within the context of geriatric specialty clinics and not within the primary care VHA population at large. Furthermore, given that the majority of geriatric Veterans are male, we cannot generalize our findings to female Veterans or to women in general; it would be interesting, for example, to observe whether the sex-related topic features in Table 2 (e.g., “wife” and “son”) varied in a more balanced sample and to determine how that may or may not affect the model’s ability to identify undiagnosed probable dementia. It was also impossible in the validation stage for our dementia specialists to definitively ascertain from clinical records alone whether every subject was “Dementia” or “Non-Dementia,” and thus, future studies may use in-person or telephone assessments to reduce that uncertainty. Likewise, because we did not directly contact the subjects who we determined were at high risk for developing dementia, we do not know how many of these patients would be amenable to additional workup and or cognitive assessments. That said, if patients are unwilling to pursue additional screening, our algorithm could still provide primary-care providers with knowledge concerning the potential risk of cognitive impairment, and it would thus encourage providers to explore alternative medical management strategies for patients who are hesitant to undergo additional assessments (e.g., routine appointments at the facility to fill medisets).

## Conclusions

In summary, our findings confirm our hypothesis that there are documented signs of dementia that can be found in all aspects of imperfect EHR data. We also demonstrate the feasibility of using our automated methods to identify topics and other EHR data that can be used to assign a dementia risk score in subjects without a previous ICD-9 diagnosis of dementia (AUC > 0.9). Our study thus suggests that there may be many Veterans with undiagnosed dementia and that by using our model, we can successfully identify these patients. These informatics advances therefore provide a striking opportunity to ultimately improve the quality of care in our nation’s aging Veterans.

## Data Availability

A minimal data set is available to authorized users of VINCI.

## Abbreviations

AD: Alzheimer’s disease
AUC: area under the receiver of characteristic curve
CCI: Charlson comorbidity index
CDW: clinical data warehouse
CPRS: Computerized Patient Record System
CPT: current procedural terminology
DSM: Diagnostic and Statistical Manual of Mental Disorders
EHR: electronic health record
eMERGE: Electronic Medical Records and Genomics Network
ICD: International Statistical Classification of Diseases and Related Health Problems
LDA: latent Dirichlet allocation
MALLET: Machine Learning for Language Toolkit
MMSE: Mini-Mental State Examination
NLP: natural language processing
ROC: receiver of characteristic
SEN: sensitivity
SPE: specificity
TSH: thyroid-stimuting hormone
VA: Veterans Affairs
VHA: Veterans Health Administration
VINCI: Veterans Affairs Informatics and Computing Infrastructure
